# Supplementary figures and images for: A dendritic guidance receptor functions in both ligand dependent and independent modes
Source: PLoS Genet. 2025 Dec 5;21(12):e1011942. doi: 10.1371/journal.pgen.1011942 (PMC12680191; doi:10.1371/journal.pgen.1011942)

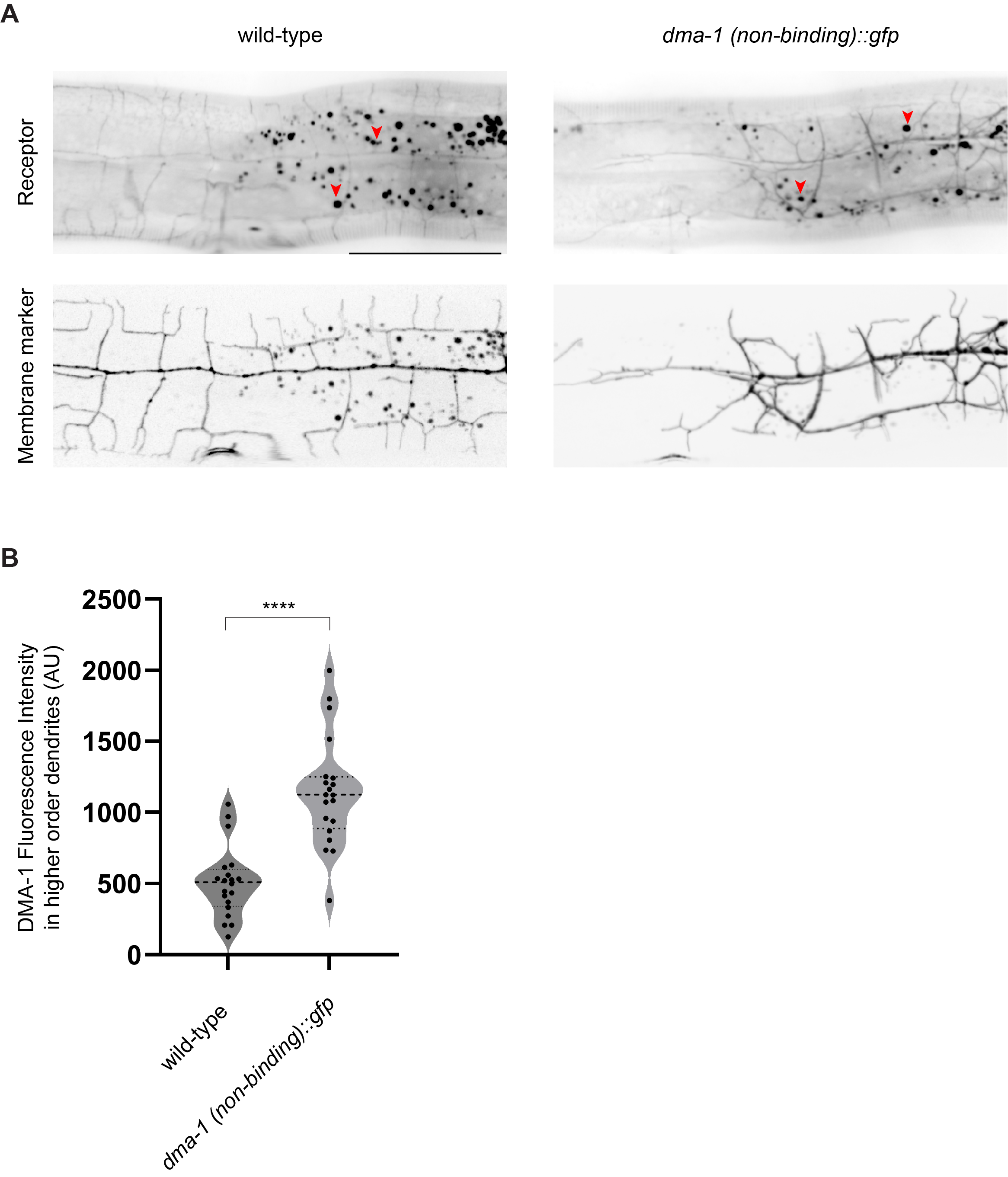

Supplement: S1 Fig — (A) (Top) Fluorescence sum intensity z-projections of endogenously labelled wild-type (left) or non-binding (right) DMA-1 receptor in Day 1 Adults. (Bottom) Fluorescence maximum intensity z-projections of PVD. Red arrowheads indicate examples of gut granules which exhibit autofluorescence and are not present in the PVD. Scale bar, 50 μm. (B) Quantification of receptor fluorescence intensity in higher order dendrites. Medians are represented in thick dashed lines and quartiles are represented in thin dashed lines. P value was calculated using a two-tailed unpaired Student’s t-test. n = 20 for all conditions. **** p < 0.0001. (TIF) [file pgen.1011942.s001.tif]

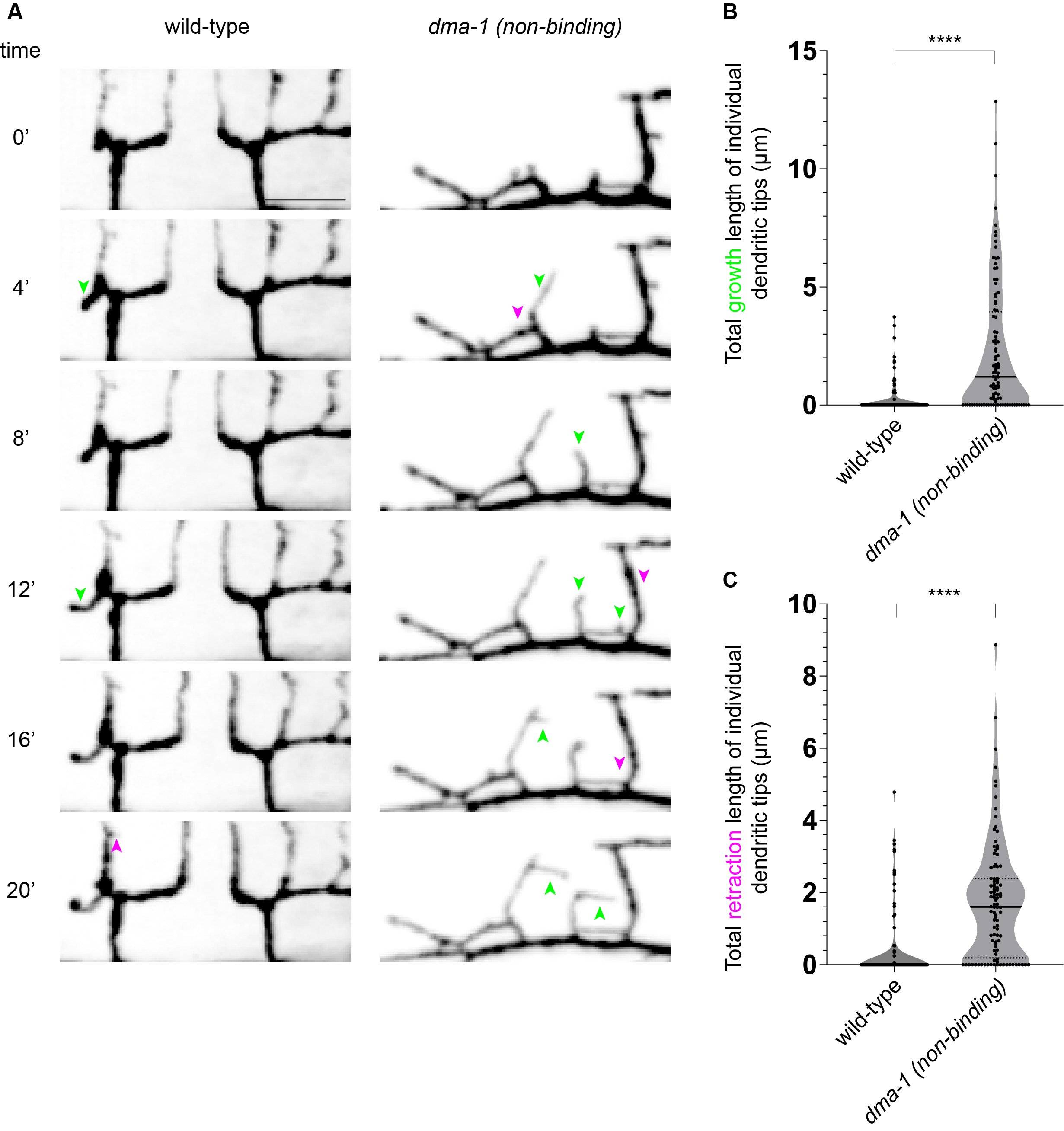

Supplement: S2 Fig — (A) Time series montage showing six frames of fluorescence maximum intensity z-projections of PVD at 4-min intervals in wild-type (left) and dma-1 (non-binding) (right) L4 animals. Growth and retraction events relative to the previous time frame are indicated with green and magenta arrowheads, respectively. Still images are from S1 Movie and represent cropped dorsal higher order dendrites. Scale bar, 10 μm. (B-C) Quantifications of total growth (B) and retraction (C) of individual dendritic tips over 20 minutes. Medians are represented in thick lines and quartiles are represented in thin dashed lines. P values were calculated using a two-tailed unpaired Student’s t-test. n = 100 for both conditions. **** p < 0.0001. (TIF) [file pgen.1011942.s002.tif]

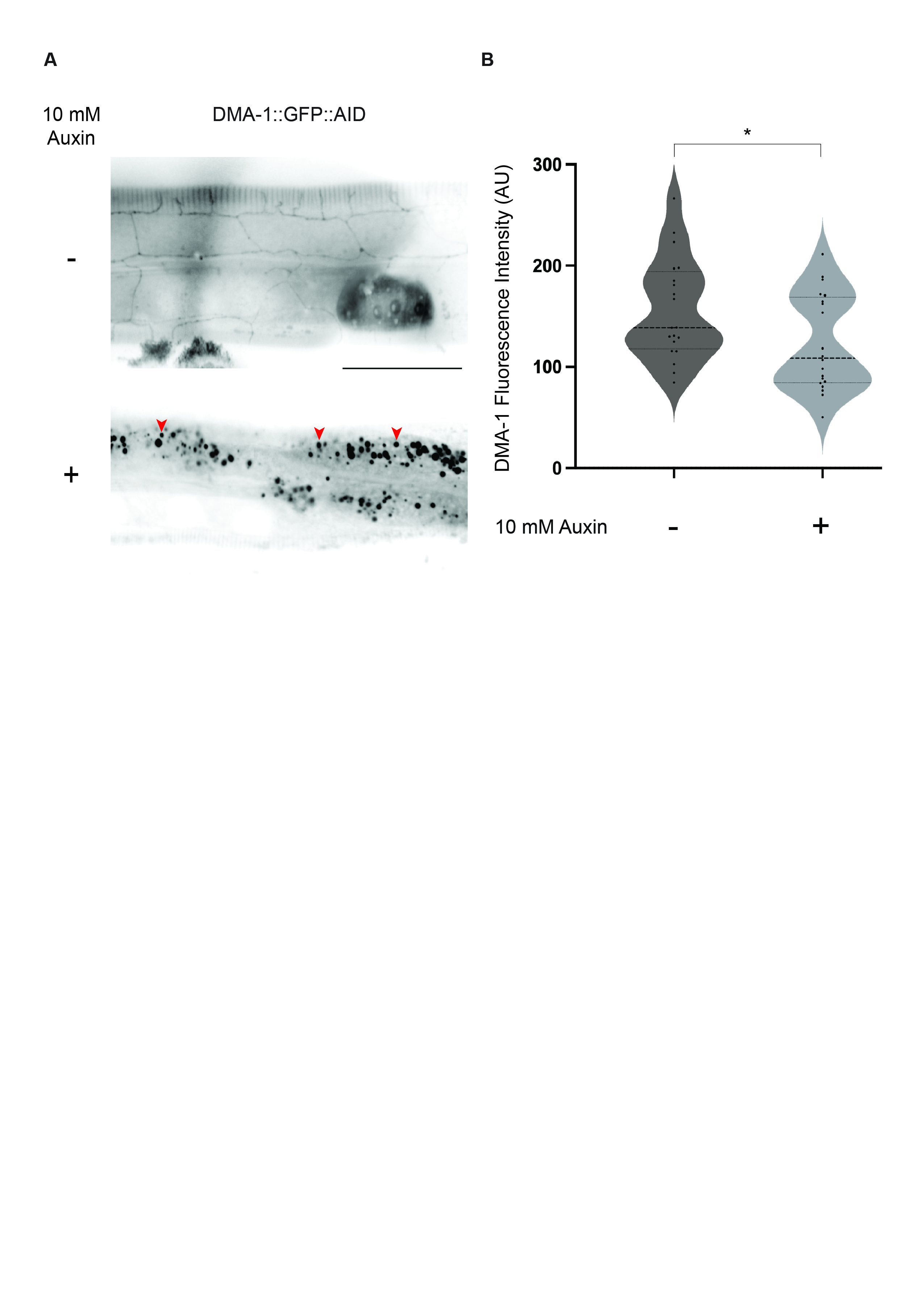

Supplement: S3 Fig — (A) Fluorescence sum intensity z-projections of endogenously labelled DMA-1::GFP::AID treated without auxin (top) or with 10 mM Auxin (bottom). Red arrowheads indicate examples of gut granules which exhibit autofluorescence and are not present in the PVD neuron. Scale bar, 50 μm. (B) Quantifications of DMA-1::GFP::AID fluorescence intensity in the PVD cell body. Medians are represented in thick dashed lines and quartiles are represented in thin dashed lines. P value was calculated using a two-tailed unpaired Student’s t-test. n = 20 for all conditions. *p ≤ 0.05. (TIF) [file pgen.1011942.s003.tif]
