# Supplementary material for: A dendritic guidance receptor functions in both ligand dependent and independent modes
Source: PLoS Genet. 2025 Dec 5;21(12):e1011942. doi: 10.1371/journal.pgen.1011942 (PMC12680191; doi:10.1371/journal.pgen.1011942)
Supplement: Attachment — Submitted filename: ResponseToReviewers.docx [file pgen.1011942.s006.docx]

Reviewer #1 The authors present their work dealing with ligand dependent and independent functions of the cell surface receptor dma-1 in PVD neuron dendrite development. They designed a non-ligand binding receptor version that is able to support dendritic outgrowth by recruiting intracellular binding partners required for actin polymerization. However, ligand binding deficient dma-1 does not support ordered dendritic development, similar to its ligand sax-7 in mutant worms. They further show that dma-1 and ligand binding is required for maintenance of already formed dendritic structures using temperature sensitive or degradable dma-1 alleles. The authors argue that the receptor is sufficient for recruiting intracellular effectors mediating dendritic growth, while structured growth and maintenance requires interaction with the ligand present in other tissues (i.e. hypodermis).
Overall, this is a very neat study showing ligand dependent and independent functions of the dma-1 receptor during development and maintenance of PVD dendrites. While the experiments are well executed and thorough, the interpretation and context with previous studies from this and other systems is very one-sided. I would urge the authors to take a more balanced view and take into account signals that regulate dendrite development differently. What they found for DMA-1 is one of many different flavors of the regulation of dendrite development.

Point #1 The dma-1 non-binding and ligand mutants showed highly disorganized dendrite growth. Can the authors comment on self-avoidance defects as dendrites seem to overlap and possibly adhere to each other extensively in the absence of ligand binding.

Response:

This reviewer’s observation is correct. Indeed, the disorganized dendrites in dma-1 (*non-binding*) and ligand mutants do not appear to avoid each other. We do not have a complete understanding of why this is the case. As the reviewer must be aware of, PVD tertiary dendrites exhibit self-avoidance behavior. While several molecules including *mig-14* and *unc-6* have been implicated in the self-avoidance mechanisms, our published and unpublished results suggest that the DMA-1/SAX-7 pathway is also involved in the self-avoidance behavior. Overexpression of DMA-1 causes strong self-avoidance phenotype of tertiary dendrites. Partial loss function alleles of sax-7, mnr-1 or kpc-1 mutants causes reduced quaternary dendrites and self-avoidance defects in 3 dendrites. We hypothesize that the contact-dependent repulsive mechanisms antagonize the DMA-1-mediated growth, and DMA-1/SAX-7 mediated stabilization. Self-avoidance is a result of the balance between these three pathways. In the absence of the DMA-1/SAX-7 adhesion mechanisms, it is conceivable that exuberant growth might override the repulsion, causing self-avoidance deficits.

Point #2 The authors labeled endogenous sax-7 with mNeonGreen and investigated alignment of dendrites with sax-7 expression (Fig. 3). I have two issues with this data: the authors point out sublateral misalignment of dendrites and sax-7 in controls, particularly of dendrites growing at a non-perpendicular angle. Is that also observed in controls without mNeonGreen-labeled sax-7 or does it indicate a partial functional impairment of sax-7? Second, the authors do not comment on the differences of the sax-7 signal in their example image. Is sax-7 indeed localized to the sublateral domain in dma-1 mutants to a similar extent as in controls? To me it seems there is only weak detectable signal and additionally intracellular accumulations suggesting sax-7 localization partially depends on dma-1 binding. Levels and/or localization should be quantified in controls vs. dma-1 mutants

Response:

To answer these questions, we compared the PVD morphology in the mNeonGreen-labeled sax-7 knockin strains and a wild type strain using the same wyIs581(ser2prom-3::mCherry) transgene. We did not find significant difference between the two strains and observed the same “slanted” secondary branches in both strains. This suggests that the mild defect is due to the mCherry transgene instead of the sax-7 knockin.

To answer the question about the sax-7 levels, we measured the knockin fluorescence signal in both wild type and dma-1 (non binding) mutants. We found no statistical difference between the two genotypes. Please see the quantifications below.

Point #3. The authors disregard prior work showing that removal of a ligand or receptor can also lead to the same phenotype and lack of dendritic patterning/growth by a failure of stabilization, e.g. instructive growth: robo/slit: PMID: 17933790, Ten-m: PMID: 24290980, NetB/fra: PMID: 21871804, permissive growth: Ret/mav: PMID: 30157422, 25764303, HSPG/RPTP: PMID: 28874572, growth/maintenance: Celsr PMID: 15296717 etc.). Instead, they refer to work where self-avoidance was analyzed dynamically showing that stochastic growth via repulsion can lead to ordered dendrite morphogenesis. This is similar in regard to stochastic growth yet distinct from the authors’ proposed mechanism presented in this study. The discussion should be more balanced in this regard.

Response:

We agree that our discussion can be more balanced. We have amended the discussion by including the following: “While we demonstrate that the DMA-1 receptor can function in a ligand-independent manner to regulate dendrite morphogenesis, other systems exhibit a clear requirement for ligand-receptor binding. For example, mutants defective for either the Robo receptor or Slit ligand exhibit similar defects in dendrite arborization of the *Drosophila* aCC motoneuron [38]. This finding suggests that the Slit ligand might promote dendrite arborization by activating the Robo receptor. Studies on the *Drosophila* class IV dendritic arborization (C4da) neurons show a similar dependency on ligand-receptor binding: both the Frazzled receptor and Netrin ligand are required for local dendrite growth towards the source of ligand [39]. Moreover, the secreted TGF-β ligand Maverick and its cognate receptor tyrosine kinase Ret both promote C4da dendrite arborization [40]. In these developmental contexts, ligand binding is required to activate an otherwise inert receptor.

There are examples of receptors functioning independently of ligand binding. Genetic evidence demonstrates that the SAX-3/Robo receptor regulates axon guidance of the *C. elegans* AWB neuron independently of its cognate ligand SLT-1/Slit [41]. Furthermore, studies in a polarized epithelial cell demonstrate that the UNC-40/DCC receptor can form clusters and recruit downstream effectors to drive F-actin formation [42]. These cases demonstrate that the activation of some receptors is not contingent on binding to a cognate ligand.” These changes are in lines 401-419.

Point #4. The authors’ model suggests that 2ndary branch growth is stochastic and then stabilized by ligand-mediated contact once reaching the sublateral region. However, typically 2ndary branches are oriented perpendicularly to the primary branch in their final configuration, suggesting that somehow the final structure is stabilized and straightened. Their model does not explain how this is achieved. Can sax-7 be released and, e.g., form a gradient or is it purely contact-dependent? For this, some level of live imaging during 2ndary branch growth is needed. The authors should also discuss other possibilities, e.g., previous work showed that mnr-1 regulates dma-1 cell surface levels and kpc-1 cleaves mnr-1 (PMID: 37721334), which might also regulate sax-7 surface levels. This study is not mentioned or discussed.

Response:

We want to thank this reviewer for bringing this up. Indeed, purified SAX-7 extracellular domains from S2 cells showed a cleaved product that was blocked by furin inhibitors (PMID: 38766073). To test whether cleaved SAX-7 promotes development of secondary dendrites at non-perpendicular angles, we imaged animals in which the SAX-7 furin cleavage site was mutated. This mutant allele showed normal branching pattern, arguing against the possibility that SAX-7 cleavage makes a major contribution to PVD dendrite branching.

We have also amended the discussion to discuss the work showing the genetic interaction between *mnr-1* and *kpc-1* as follows: “This model can also explain the genetic interaction between the partial loss of *kpc-1* and partial loss of *mnr-1*: the truncated tertiary dendrite morphology of *kpc-1* mutants can be partially rescued by lowering MNR-1 dosage [37]. In *kpc-1* mutants, plasma membrane localized DMA-1 increases whereas endosomal DMA-1 decreases [27]. It is possible that when KPC-1 activity is attenuated, the lowering of functional MNR-1 both reduces the adhesion of plasma membrane localized DMA-1 and promotes receptor endocytosis. This rebalancing of the two pools of DMA-1 enables the partial rescue of the truncated tertiary dendrites.” Please see lines 392-400 in our manuscript.

Point #5. The degradable dma-1 allele (as well as the ts allele at 16°C) seem to be only partially functional based on the authors’ analysis of 4° branches. This should be clearly mentioned as this represents an already sensitized background that is not fully comparable to a wild type situation.

Response:

The reviewer is indeed correct in noting that the severity of higher order branch loss of *dma-1* (*ts*) at 25°C appears to be partially functional and is a sensitized background. We have made a slight modification to Fig 5d to compare wild-type and *dma-1* (*ts*) quaternary number at permissive temperatures. The original version incorrectly compared wild-type animals grown at restrictive temperatures to *dma-1* (*ts*) grown at permissive temperatures. We have also amended the text to clearly state that the *dma-1* (*ts*) allele is a partial loss-of-function.

Minor:
1. The authors cite numerous reviews related to their work but little primary references. This should be amended for the main points of their interpretation.

We agree that our work needed to cite primary references when discussing molecular mechanisms driving dendrite morphogenesis. We have included the following primary references: PMID: 15608632, PMID: 21423166, and PMID: 14578357.
2. The first chapter of results is basically a summary of known findings based on the previous work of the authors and others. Please move this to the introduction.

We have moved this to the introduction. Please see lines 109-135.

**Reviewer #2**: In this manuscript, Reddy et al. identified three critical residues in the extracellular domain of the DMA-1 dendrite guidance receptor, and showed that these mutations abolish the binding between DMA-1 and the dendrite ligand complex in vitro and proper PVD dendrite morphogenesis in vivo. Using confocal imaging, they compared the PVD dendrite morphologies in dma-1 null, ligand null and dma-1 (non-binding) mutant strains, and found that dma-1 (non-binding) mutant animals phenocopy ligand null mutants. In addition, dendrites can grow with or without SAX-7 signals, suggesting that PVD dendrite can grow in a ligand complex-independent manner. Genetic double mutant analyses showed that dendrite outgrowth in the dma-1 (non-binding) mutants relies on HPO-30 and TIAM-1, which are critical regulators for high-ordered branch formation in normal menorah formation as reported previously. The authors further used a temperature-sensitive dma-1 allele and auxin-induced protein degradation assay, and nicely showed that DMA-1 and the receptor-ligand interaction are required for dendrite adhesion/stabilization. Overall, the quality of most results is high, and most of the conclusions are well supported by their data. A few additional experiments may strengthen the quality of this study.

Point #1. In Figure 1, the authors performed a SPR assay to show that a mutant form of DMA-1 ECD (R106D-S107A-R109E) completely abolishes the receptor-ligand interaction in vitro. This is a very nice assay using purified proteins. However, it seems that they cannot perform these experiments using endogenously expressed full length proteins due to technical issues. As each of DMA-1, SAX-7 and MNR-1 contains a single transmembrane domain, it is critical to use alternative assays to test whether the three mutations really abolish the receptor-ligand binding (ideally in vivo). Thus, it would be nice for the authors to test this using the single molecule pull-down and the fly S2 cell aggregation assays, which were successfully used in their previous studies (PMID: 24120131; PMID: 27705746; PMID: 29738713).

Response:

Compared to the SPR assay, both single molecule pull-down and S2 cell aggregation assays are far less quantitative. It is also harder to be sure about the protein folding impacts in the single molecule pull down or S2 cell assays, especially if the *dma-1* mutants are likely to yield a non-binding result. Given the identical dendrite phenotypes between the dma-1(R106D-S107A-R109E) and the ligand null mutants, we are confident that the mutations have disrupted the ligand-receptor interaction. In a parallel study that we are preparing, Engin Ozkan’s group has solved the structure of the ligand receptor complex. We have done additional mutagenesis experiments to test the validity of the structure. The results are consistent with the AlphaFold prediction.

Point #2. Did the authors examine whether the three mutations (R106D-S107A-R109E) affect endogenous DMA-1 folding, trafficking or localization? This could be done by tagging both the WT and mutant form of DMA-1 with GFP using CRISPR and then perform confocal imaging.
Response:

This is an excellent suggestion that Reviewer #3 also asked about. We introduced the three amino acids into an endogenously tagged *dma-1::gfp* allele and compared fluorescence intensity of wild-type and mutant receptors. We have previously shown that mutants that perturb ER protein folding, such as the *ire*-*1* mutant, causes DMA-1 to be largely reduced in the dendrite and instead accumulates in the somatic ER (PMID: 39229034). In the dma-1 (R106D-S107A-R109E)::GFP knockin strain, we found that mutant receptor is not trapped in the soma but instead robustly localizes to higher order dendrites, indicative of normal folding of mutant receptors. Interestingly, we noticed that the level of mutant receptor at the dendrites was greater than the wild-type receptor. This is consistent with a finding that wild-type receptor is enriched at the membrane in *sax-*7 mutants (PMID: 38766073). The enhanced level of membrane DMA-1 is likely due to decreased receptor endocytosis, which is dependent on ligand-receptor binding. These findings argue against the possibility that the introduced mutations cause defects in receptor folding or trafficking. We have represented these data in S1 Fig. For textual changes, please see lines 209-217.

Point #3. In Figure 2, they imaged PVD dendrite morphologies in both WT and mutant animals and found that dma-1(non-binding) mutants phenocopy sax-7(null), as they generated more and longer dendrites when compared with the dma-1 null mutant animals. What is the underlying mechanism? This phenotype in dma-1(non-binding) could be explained by either increased outgrowth activity or reduced retraction or both, which can only be tested using time-lapse recording analysis. Although DMA-1 (non-binding) cannot bind with the SAX-7-MNR-1-LECT-2 ligand complex as supported by the SPR assay, it possibly binds to a yet-identified ligand. Thus, it would be nice if they can perform time-lapse analyses for WT and all the receptor/ligand mutants tested here, and add dma-1 delta ECD (reported in PMID: 38766073) as a control.

Response:

This is another excellent suggestion which we have incorporated in our resubmission. We performed time-lapse fluorescence microscopy on wild-type and *dma-1 (non-binding)* L4 animals to assess dendrite dynamicity. We chose this developmental stage because wild-type animals will still exhibit growth events, as opposed to young adult animals which have completed their growth. To measure growth and retraction, we quantified the length of individual dendritic tips at each time point and measured the change of length between each time point. We quantified the total growth and retraction length of individual dendritic tips and represented the results in S2 Fig b, c and S1 movie.

Our findings demonstrate that the phenotype observed in *dma-1 (non-binding)* animals arises from mutant dendrites having both increased growth and increased retraction events when compared to wild-type. These data complement our later findings in the manuscript, where we demonstrate that ligand-receptor binding is required for stabilization of higher order dendrites. For the textual edits, please see lines 276-282.

Point #4. The authors used both a dma-1(ts) allele and an auxin-induced protein degradation assay to test whether DMA-1 is required for branch stabilization, and whether the receptor-ligand binding is required. In Figure 5, the number of tertiary and quaternary branches were quantified. However, in Figure 6, only the number of the quaternary branches were quantified. It seems to this reviewer that the severity of the dma-1 (ts) mutants in 25°C is more severe than the dma-1-gfp-aid animals with auxin. Is that true? If so, why is that? For the results showed in Figure 6, is the receptor-ligand interaction specifically required for the stabilization of quaternary branches, but not for secondary or tertiary ones? Based on the results showed in S1 Fig, it seems that the auxin-induced degradation of endogenous DMA-1 is robust. Is it possible to measure the DMA-1 degradation efficiency using an alternative assay such as western blotting? In addition, could the author explain why dma-1::gfp::aid/+ animals grow more quaternary branches than the dma-1::gfp::aid animals do?

Response:

The reviewer raises several excellent points here, which we will address in detail. Firstly, the reviewer is indeed correct in noting that the severity of higher order branch loss of *dma-1 (ts)* at 25°C appears to be more severe than the *dma-1::gfp::aid* animals treated with auxin. In support of this, we observed defects in tertiary branch formation upon temporal downregulation of *dma-1 (ts)*. We have amended the text to clearly state that the *dma-1 (ts)* allele is a partial loss-of-function. Secondly, the reviewer is correct in their speculation that we did not observe loss of secondary or tertiary branches upon auxin treatment of animals containing a *dma-1::gfp::aid* allele. While the reviewer is also correct in stating that the degradation of degron tagged receptor is indeed robust, as reflected in Fig S3, this does not necessarily imply that the degradation is complete. To expand on this, we attempted a similar experiment to test the requirement of ligand-receptor binding for the maintenance of higher order dendrites by tagging the SAX-7 ligand with an mNeonGreen sequence fused to the AID degron. While fluorescence microscopy clearly demonstrated robust degradation of SAX-7, this degradation did not lead to disordered dendrite arborization which is observed in *sax-7* (*null)* animals. This example raises the possibility that our temporal downregulation of *dma-1::gfp::aid* was sufficient to perturb quaternary formation but may not have been complete enough to degrade all of DMA-1. The remaining DMA-1 may be sufficient to maintain secondary and tertiary branches. Thirdly, the reviewer asks why animals containing one copy of the wild-type receptor allele grow more quaternaries than *dma-1::gfp::aid*. Interestingly, this is also observed in the absence of auxin treatment. We speculate that the degron tagged receptor is undergoing leaky degradation, which has been observed previously with other degron tagged proteins (PMID: 34865044). Therefore, it is likely that the *dma-1::gfp::aid* allele is a weak loss-of-function allele in the absence of auxin.

Point #5. To provide additional evidence to demonstrate that DMA-1(R106D-S107A-R109E) specifically affects dendrite stabilization but not outgrowth, the authors may test whether their dma-1(non-binding) mutations affect seam cell trapping induced by ectopically expressed SAX-7 in the seam cells (as reported in PMID: 24120131 and PMID: 24120132).

Response:

The seam cell trapping phenotype is dependent on *dma-1* because the *dma-1(null)* suppress the dendritic trapping with reduced dendrite growth. As a result, the DMA-1(R106D-S107A-R109E) experiment by itself might not be the best to distinguish whether DMA-1(R106D-S107A-R109E) represent simply a complete loss of function, partial loss of function or loss of a specific function. In every other aspect of our characterization, DMA-1(R106D-S107A-R109E) is indistinguishable from the ligand mutants. While we believe the reviewer’s experiment is certainly feasible, we believe our existing experiments have shown this point and therefore did not pursue this specific experiment.

Reviewer #3. In this manuscript, Reddy and colleagues, investigate the mechanism of how the leucine-rich transmembrane receptor DMA-1 in PVD functions in dendrite morphogenesis. Prior work had established that DMA-1, and its ligands SAX-7, MNR-1, and LECT-2 are required for patterning of PVD somatosensory dendrites. Early on, it was noted that the mutant phenotypes of DMA-1 null mutants were more severe than the ligand mutants suggesting that DMA-1 may serve ligand independent functions. This is observation is the premise for the current manuscript. Using Alphafold, the authors model the structure of the SAX-7/DMA-1 complex and identify three key residues in DMA-1 predicted to constitute key residues for the molecular interaction of the cell adhesion molecule SAX-7 and DMA-1. Biochemical experiments using Surface Plasmon Resonance establish that indeed these three residues are key for the SAX-7/DMA-1 interaction in vitro. Consistent with the hypothesis, the authors further find that introducing these three point mutants into the DMA-1 locus results in phenotypes that are indistinguishable from the ligand mutants SAX-7, MNR-1, and LECT-2 (ie less severe than the DMA-1 null mutants). Additionally, the dendrites of the non-binding DMA-1 allele fails to colocalize with a SAX-7 reporter. These findings are consistent with the interpretation that DMA-1 binds to SAX-7 in the same manner in vivo. The authors then go on to show that both ligand-dependent and ligand-independent DMA-1 function through the same downstream mechanisms, based on the observation that mutations in the downstream effectors appear epistatic to the DMA-1 non-binding mutant. The manuscript ends with data suggesting that DMA-1 ligand binding is important for dendrite stability and maintenance. Overall, this is a well written paper that adds an important new facet to the understanding of dendrite patterning. The genetic and biochemical experiments are rigorous and well executed and generally support the conclusions. I have some comments that should be addressed:

Point #1. The biochemical and genetic experiments are consistent with the model the authors propose, namely that ligand-free action of DMA-1 is sufficient for (stochastic) outgrowth and that ligand binding leads to stabilization of the processes. To further support their model, the authors should test whether an endogenous DMA-1 reporter harboring the three point mutations that abolish binding to SAX-7 shows similar staining patterns as a wild type DMA-1 reporter. This experiment would exclude that trafficking or misfolding of the DMA-1 receptor are responsible for the partial loss of function phenotype, a possibility the authors do not entertain.

Response:

We introduced the three amino acids into an endogenously tagged *dma-1::gfp* allele and compared fluorescence intensity of wild-type and mutant receptors. We have previously shown that mutants that perturb ER protein folding, such as the *ire*-*1* mutant, causes DMA-1 to be largely reduced in the dendrite and instead accumulates in the somatic ER (PMID: 39229034). In the dma-1 (R106D-S107A-R109E)::GFP knockin strain, we found that mutant receptor is not trapped in the soma but instead robustly localizes to higher order dendrites, indicative of normal folding of mutant receptors. Interestingly, we noticed that the level of mutant receptor at the dendrites was greater than the wild-type receptor. This is consistent with a finding that wild-type receptor is enriched at the membrane in *sax-*7 mutants (PMID: 38766073). The enhanced level of membrane DMA-1 is likely due to decreased receptor endocytosis, which is dependent on ligand-receptor binding. These findings argue against the possibility that the introduced mutations cause defects in receptor folding or trafficking. We have represented these data in S1 Fig. For textual changes, please see lines 209-217.

Point #2. The authors should consistently write the vertebrate homologs with the C elegans gene names to make the manuscript more accessible to a vertebrate readership.

Response:

Thank you for the suggestion. We have included the vertebrate homologs for the genes *lect-2* (LECT2), *sax-7* (L1CAM), *mnr-1* (FAM151b), and *tiam-1* (TIAM1). However, the genes *dma-1* and *hpo-30* lack a clear vertebrate homolog based on primary sequences. While a recent review written by Max Heiman and Hannes Buelow (PMID: 38785371) depicted the homologs of *dma-1* and *hpo-*30 as LRR-TM and Claudin respectively, we felt like these were families of genes instead of specific vertebrate homologs. Therefore, we did not include homolog names for these genes.

Point #3. Are there potentially analogous findings in the vertebrate literature where receptor knockouts are more severe than ligand knockouts, where similar mechanisms could be at play? If so, that may be worth mentioning to broaden the significance.

Response:

We thank the reviewer for this suggestion. We have now added a more detailed discussion about other examples of ligand receptor discrepancy (lines 413-419). “There are examples of receptors functioning independently of ligand binding. Genetic evidence demonstrates that the SAX-3/Robo receptor regulates axon guidance of the *C. elegans* AWB neuron independently of its cognate ligand SLT-1/Slit [41]. Furthermore, studies in a polarized epithelial cell demonstrate that the UNC-40/DCC receptor can form clusters and recruit downstream effectors to drive F-actin formation without binding its cognate ligand UNC-6/netrin[42]. These cases are consistent with the idea that some receptors might have ligand independent functions.”

Both examples come from *C. elegans*. In vertebrate systems, there are usually several paralogs such as ROBO1-3 and slit1-3, so therefore it becomes more difficult to create compound mutants to knockout all ligands.

Point #4. The authors state that the lect-2; mnr-1; sax-7 triple null animals exhibit a slightly increased number of quaternaries when compared to the sax-7 (null) and dma-1 (non-binding). I don’t see this in the data (only high order branches are shown) or did I miss it? Could the authors clarify and if true, what would the authors make of this observation?

Response:

In our original manuscript, we included a comparison in Fig 2i showing that the number of quaternaries in the triple null mutant was slightly higher than *dma-1 (non-binding)* and *sax-7 (null)*. In our scoring, we defined a quaternary dendrite that branched from a tertiary in an orientation that was away from the primary dendrite. This scoring criteria effectively captures the vast difference in quaternary number between wild-type, which display numerous and robust quaternaries, and mutant classes, which display sparse and poorly formed quaternaries. However, given the poorer quality of mutant quaternaries, this criterion is not as effective at evaluating differences in quaternaries between the mutant classes. Instead, a far superior comparison between mutants is that of the total higher order dendrite length, which is captured in Fig 2j.

The reviewer’s comment brought to our attention the possibility that this comparison may distract the audience from the main point of Figure 2: that in terms of dendrite outgrowth, the *dma-1 (non-binding*) mutant resembles ligand null mutant classes but is clearly distinguishable from *dma-1 (null)*. Therefore, regarding the comparison of quaternary number between mutant classes, we have removed this comparison from Fig 2i and have removed the text (originally in lines 213-216 in our first submission) explaining the difference.

Point #5. Somewhat confusing in the author’s model is the fact that the same downstream signaling mechanisms are employed by ligand dependent and ligand-independent functions of DMA-1? Something must be different between the two. What is it, if not the downstream signaling molecules? Could the authors at least speculate?

Response:

We apologize for the confusion. Our data show that the ligand free DMA-1 requires HPO-30 and TIAM-1 for its activity to promote disorganized dendrite growth. We do not have evidence that suggest that HPO-30 or TIAM-1 is required for the ligand bound DMA-1’s function in stabilizing the dendrites. We have not succeeded in generating conditional alleles of *hpo-30* and *tiam-1* to test this idea. Both genes are required for the growth of PVD dendrites, therefore, we need to conditionally delete these genes after dendrite growth and demonstrate that we can completely deplete the proteins. To avoid confusion, we have made changes to Fig 7 and in the text to better reflect this. For the textual changes, please see line 241 and a modification made at line 850.

Point #6. The structure of MNR-1 is conspicuously absent from the model. Why?

Response:

We thank this reviewer for pointing out that the structure of MNR-1 is missing. Given that Alphafold 2 predicted that the identified receptor residues were critical for binding the SAX-7 ligand, we only showed the structure between DMA-1 and SAX-7/L1CAM in Fig 1c of our original submission. To fix this, we have submitted an updated version of this figure in which we show the Alphafold 2 prediction of the entire tripartite ligand complex and the DMA-1 receptor in panel c. Furthermore, we have modified our reporting of the iPTM value in the Materials and Methods. For the textual changes, please see line 448.

Minor edits:

Line 99: underline after a period.
Line 139: PVD was described essentially concomitantly by three labs: Oren-Suissa et al 2010 (PMID: 20448153), Smith et al. 2010 (PMID: 20537990) and Albeg et al. 2011 (PMID: 20971193). All three papers should be cited here.

Line 183: does the SAX-7 ectodomain bind to DMA-1 in the absence of fused MNR-1?
Line 259: while the authors make a correct conclusion here, both hpo-30 and tiam-1 could also be acting in parallel. This must be mentioned as a possibility.
Line 275: …whether DMA-1 has A role

Thank you for pointing out these mistakes. We have made the following changes in the revised manuscript:

Line 98: We have removed the underline after the period (originally line 99 in the first submission).

Line 260-261: We have mentioned the possibility that HPO-30 and TIAM-1 may function in parallel to promote outgrowth driven by ligand-free receptor (originally line 259 in the first submission)

Line 283: We have rewritten this line, and the grammatical mistake has been corrected (originally line 275 in the first submission).
